# Physiology of War

**Published:** 1864-05

**Authors:** 


### HALL'S JOURNAL OF HEALTH.

Our Legitimate Scope is almost boundless: for whatever begets pleasurable and harmless feelings, promotes Health; and whatever induces disagreeable sensations, engenders Disease.

WE AIM TO SHOW HOW DISEASE MAY BE AVOIDED, AND THAT IT IS BEST, WHEN SICKNESS COMES, TO TAKE NO MEDICINE WITHOUT CONSULTING A PHYSICIAN.

Vol. XI.] MAY, 1864. [No. 5.

### PHYSIOLOGY OF WAR.

GOING down Broadway any day, scores of men and women may be observed as unconscious of the presence of their fellow-men, despite the pressing along of the ceaseless crowd, the rattling of wheels, and the din of business, as if they were in the midst of Sahara or some boundless prairie of the West, or in an Indian canoe in mid ocean. There may be noticed at any hour, the compressed lip, the muttering speech, the sharp exclamation, the impatient gesture, and the smothered curse. The public pulse beats fast and high and hard; the machinery of life is running at a rate so abnormal in its rapidity, that it must wear out long before its time, or be shivered to atoms by the unnatural tension.

"Died suddenly," is the frequent announcement of the morning paper. "Who died suddenly?" The merchant whom we met on 'Change not thirty-six hours ago; the broker whom we saw on the street yesterday noon with flushed face, and fingers clenching a package of papers, the loose ends of which were fluttering in the wind. He was on the half-run, to get into his grave, and there he is. Go into a man's office, he does not ask you to take a seat; that would imply that some long story was to be listened to; he forgets to say good-morning, and with inquiring look he asks you to begin what you have to say, and be off. The visitor is just as intent on business as the visited, and the first sentence, sometimes the first word and only word indicates the whole object of the interview. A man calls at the post-office for a letter: "Good-morning, neighbor, fine day to-day; all well at home? I called to see if there was any letter for me to-day, as I was expecting one." Does he make all this ado? Why, the post-office clerk would faint away; he wouldn't get through his work till midnight. You appear at the window, announce your name, the pile is looked over, the letter is silently handed to you, or the monosyllable "none" is uttered; you give room for the impatient man behind you, and all is over. The minister would be considered an old fogy who would hum and haw and beat around the bush twenty or even ten minutes, as in the olden time, before he announced the subject-matter of his discourse. He is expected to present the main idea in the first sentence, and without more ado, present his divisions, offer his proofs, make the application, and away, all in forty minutes, and wiser they who do it in thirty; beyond forty he becomes tedious, is unheard; irritation springs up, and he is pronounced "repetitious."

A man enters the breakfast-room with one arm in the sleeve of his coat, the other half-way; gobbles down his coffee and toast and tenderloin in silence, grabs up the morning paper, and at a two-forty gait makes for the car or omnibus, and is oblivious to all the world until he reaches his destination.

Said a thoughtful wife the other day: "My husband never thinks of his dinner; if I put a sandwich in his pocket in the morning at seven, it is there still when he reaches home at six; eleven hours, not a mouthful eaten; business, business, business!"

"My husband didn't sleep two hours last night," said a charming woman not long ago. "I waked up, and in the full glare of gas-light he was pacing the floor, and continued it until the morning." "Nor does mine sleep," said another wife, whose husband is one of the men of the time. "He tosses and tumbles the whole night through, and merely dozes for an hour."

"Three hours is all the sleep I can get in the twenty-four," said a man of great wealth, the other day. "I would be willing to begin where I began before, a poor boy, without a penny in the world, if I could sleep as I did then."

But there are moral aspects of the war more astounding, and still more to be lamented; it is the perfect breakdown of all personal morality. There is a recklessness of moral principle pervading all classes, (individual exceptions everywhere,) which almost makes the thoughtful feel that the millennium has been indefinitely postponed. Deception, extravagance, recklessness, and waste are everywhere in the ascendant, except in families long rich. The servant and the master; the employers and the employed; the boss and the journeyman; the apprentice and his teacher; all, all seem half demented; seem to act as if gold grew on every twig, and want was never to be known again.

But with all our admiration of womankind, it must be confessed that the wives and daughters of the common and aspiring classes are running riot in their fierce madness after fine dress, showy equipages and splendid mansions; few among these know the value of money, and fewer still care whence or how it comes, so they can get it with the trouble of asking, quarreling or crying for it. Not one in a thousand of them appreciates the risks, and toils, and vexations and crushing responsibilities involved on the part of their husbands in providing for their households in times like the present. Formerly a man could do business with his next-door neighbor without fear or misgiving, but the moral sense is so obtunded now that fellow can not trust fellow, nor friend, friend. To trust is to be defrauded. To favor, is to lose all.

Men who formerly stood high among their fellows in New-York, Philadelphia, and Boston, have become government contractors and have been proved to be unprincipled scoundrels. Broadway clothing stores have furnished rotten coats to poor and suffering solders; ship-brokers have made out of the government scores of thousands in an hour. Money makes all laws, and unmakes them, as witness the whisky tax in Congress; the tobacco tax, the efforts to remove the duty from paper and coal; in a thousand other directions in Washington, in Albany, in Harrisburgh, and above all, in Trenton, corruption and trickery are the order of the day.

The monetary affairs of the country are rapidly verging to a common ruin. Money is apparently plenty, but never has it been so scarce in the history of the government. Coin is the only money, and nine persons out of ten fail to receive or pay out a five-cent piece in a month's traffic. Never were silk and satin and velvet so high in price; never were they seen so common on the street as at the present time.

War presents some curious features to our view. It has drained our cities in large part of a redundant, idle, diseased and degraded class; these either soon die or are killed off. But there are examples not a few where the activities of the camp, its discipline and its experience have made invalids robust; have imparted a higher moral tone to some, and given character and energy to others, who before were by common consent considered to be inane and worthless.

When a man of a good common education and some steadiness of character, goes to war and fairly engages in battle, he is thereafter, until his dying day, more of a man than he ever was before. No one of even common observation can have failed to notice in the faces of returned veteran regiments as they have marched along our streets, a stereotyped cast of countenance, common to all; there is an imprint of sternness on every face; of determination, and an elevation of spirit, despite of tattered garments and soiled clothing and the dust and sweat of a long march; as much as to say, I have been fighting for my country, I have imperiled my life to maintain her liberties and her unity; these are first things; my mission is god-like, to wit, to maintain liberty and the right forever! Amen.

When this war is ended, much of the scuff and scum of society will have disappeared, and nine out of ten of those who return from victorious battle-fields will make better, sterner, more manly members of society than ever before. The most of the great soldiers of history were men of simple tastes, quiet manners and of unassuming deportment. This is the tendency of war, to lop off excrescences, to consolidate the character, to inure to self-denial, to impart energy, determination and self-reliance, and to mold the whole man aright. This war will leave more men in the country than were found in it the day when Sumter was fired at and fell.

Official reports of European countries have shown more boy-children are born in war than in times of peace, and that although at the end of the wars of the First Napoleon, it was rare to find a Frenchman over five feet three, there was a recuperation in the next age, and now the average hight of the men does not vary much from what it was before the Directory.

As soon as the war closes there will inevitably be a universal financial crash; in five years thereafter the country will exhibit a degree of solid prosperity and national power which can defy the world besides; an amount of cotton will be raised annually, which will astonish all civilized nations. Why?

War makes men; determined, self-reliant men; such men have a degree of self-respect which idlers never dreamed of; these characteristics will impel them to labor; to intelligent labor, to labor well directed. Five years ago, many a planter had from five hundred to five thousand acres of land, of which a few hundred only were cultivated, the remainder was held in reserve for children who were growing up with the expectation of a fortune and with the full calculation to live in ease and luxury, to end in a life of idleness, intemperance, and debauchery. Five years hence, there will be ten households instead of one, to every thousand acres; there will be ten families instead of one to be supplied with school-books, and libraries; with the ubiquitous newspaper; the weekly journal and the monthly magazine. Ten families will want a sewing-machine, a piano, a reaper and a clothes-wringer, where one does now. Ten neat cottages will spring up, where was seen but five years since a solitary planter's house, never papered, seldom plastered, and always in a more or less unfinished condition. Intelligence will not plant the teeming soil with corn and potatoes at a price of twenty dollars an acre when it can raise a hundred dollars' worth of cotton, and sometimes three hundred dollars' worth, with less labor.

That country is strongest, is most prosperous, and can best defy all outside nations which is marked off into farms of forty, fifty, or an hundred acres instead of embracing ten or twenty of these in one partially tilled plantation. So that aside from the mere question of slavery there will be benefits arising from this war which will present an encouraging front compared with the opposite phases.

The ravage of war as to human life is exaggerated in almost all minds, and is never so great as it seems to be. Many of the soldiers who sicken and die in hospitals would have sickened and died at home; while the proportion of all who die from wounds is astonishingly small, and some of these would have perished by accident had they remained at home.

It can not be denied that war is always a curse; and can seldom, if ever, fail to be a sin; but as in the present state of human morals it will come sooner or later, to the nationalities of the earth, it is well to look at both sides calmly and dispassionately, take an intelligent view of all its phases, and endeavor to make the best of it.

